# Validating the decent work scale incorporated with a social recognition component among young adult social workers

**DOI:** 10.3389/fpsyg.2022.985664

**Published:** 2022-09-28

**Authors:** Xuebing Su, Victor Wong, Kun Liang

**Affiliations:** ^1^Department of Applied Social Sciences, The Hong Kong Polytechnic University, Kowloon, Hong Kong SAR, China; ^2^Department of Social Work, Hong Kong Baptist University, Kowloon, Hong Kong, SAR China; ^3^Department of Social Work, East China University of Science and Technology, Shanghai, China

**Keywords:** decent work, psychology of working theory, social recognition, sustainable development, social workers, helping professionals, psychosocial perspective, youth development

## Abstract

The decent work notion has sparkled a keen academic interest in studying the psychological influence of decent work on workers in organizational contexts. Duffy’s decent work notion has left a window for addressing the interpersonal barriers on or factors for enhancing people’s equal access to decent work, which may enhance the capacity of the decent work notion and the psychology of working theory to promote inclusiveness within the organizational context through leveraging the interpersonal mechanisms. Against this backdrop, a across-sectional study was conducted to validate a decent work scale incorporated with a social recognition component among young adult social workers aged 21–29 in Hong Kong (*N* = 362). The results of confirmatory factor analyses supported the six-factor-higher-order model of the decent work scale incorporated with a social recognition component. Decent work incorporated with social recognition correlated with job demands, job resources, and work engagement in the expected directions, and the results of average variance extracted analyses supported the discriminant validity of the decent work scale incorporated with social recognition. The value added by decent work in enhancing work engagement after controlling the effects of job resources justifies the concurrent validity of the concept. The expanded notion of decent work incorporated with the social recognition component is deemed applicable to informing further research and practice.

## Introduction

Decent work as an important concept and a global agenda for promoting social justice in social, political, and economic development has attracted increasing academic and policy interest in the past 20 years (Bellace, 2011; Silva, 2021). The term decent work was firstly initiated by the (International Labor Organization (ILO) in 1999), which was later used to guide the assessments of working conditions at macrolevel that can be counted as decent including union density, occupational safety, legal protection for workers, availability of social security, etc. (International Labor Organization, 2012). Nowadays, the term decent work has been promoted all over the world at theoretical, practical, and research levels (Pereira et al., 2019; Rantanen et al., 2020) for the sake of achieving the four main values underlying the ILO’s actions, namely freedom, equity, security, and human dignity, which are specified in the Decent Work Agenda (DWA; International Labor Organization, 1999:3). The decent work notion originated in western societies has been introduced into non-western contexts such as China since the early 2000s at different levels as well (Cooke et al., 2019; Yang et al., 2019).

Informed by the psychological perspective, Duffy et al. (2016, 2017) developed the Decent Work Scale consisting of five factors, namely safe working conditions, access to health care, adequate compensation, free time and rest, and complementary values. Some recent empirical studies (Di Fabio and Kenny, 2019; Masdonati et al., 2019; Ribeiro et al., 2019; Vignoli et al., 2020) confirmed the importance of Duffy’s five factors with the support of quantitative findings, but also sounded out the importance to expand the conceptualization and operationalization of decent work with reference to social contexts. The conceptualization and operationalization of the decent work notion is still evolving with respect to expanding the capacity of the concept in enhancing positive work outcomes and promoting inclusionary employment (Ferraro et al., 2018; Seubert et al., 2021). With an emphasis placed on individual’s psychological perception and assessment of work conditions, the psychological perspective underlying Duffy’s conceptualization and operationalization of decent work (Duffy et al., 2016, 2017) has left a window for using a psychosocial perspective characterized by an interpersonal or relational angle for conducting research studies, which may help pinpoint the negative effects of social barriers such as discrimination and marginalization on people’s equal access to decent work, and address the importance of interpersonal or psychosocial mechanisms for reshaping people’s experience of work (Rossier and Ouedraogo, 2021; Seubert et al., 2021). To address the psychosocial barriers encountered by people at work may expand the capacity of the decent work notion with regard to promoting workplace wellbeing and work fulfillment on both research and policy-making levels through leveraging interpersonal or intersubjective mechanisms. Moreover, there has emerged a growing concern to speed up the review of decent work for caring about the benefits of service workers such as domestic workers, hospitality workers, and social workers, whose level of workplace wellbeing is deemed highly associated with their misrecognition or under-recognition experiences, and this concern is suggesting to incorporate social recognition as a component into the decent work notion (Chen, 2011; Charlesworth and Malone, 2017; Tiemeni, 2018; Molina et al., 2020). Informed by Honneth’s recognition theory, this study aims to validate the Decent Work Scale incorporated with a social recognition component among young adult social workers.

### Expanding the decent work notion by incorporating a social recognition component

Using a sample of fifty workers in Ouagadougou, the capital of Burkina Faso, Rossier and Ouedraogo (2021) conducted a preliminary study to validate the Decent Work Scale with three additional items (i.e., “I’m doing work that is clean,” “I’m doing work that is honest,” and “I’m doing work that brings me social value”) which were used to measure social recognition. Yet, this study did not provide a clear definition of social recognition and the three items used to measure social recognition did not explicitly address the role played by interpersonal or intersubjective dynamics. Without such a perspective, these items are less likely to advocate interpersonal changes at workplace for promoting equal access to decent work. More theoretical and empirical work to validate the Decent Work Scale with a recognition component is needed.

Informed by the recognition theory developed by Honneth (1995, 2001, 2012), we define social recognition as the need for mutual acknowledgment within a given context. In the workplace context, social recognition refers to people’s need to strive for mutual acknowledgment from their supervisors and colleagues, which may come in the form of care, respect of rights and agency, encouragement, appreciation, etc. Honneth’s recognition theory highlights an “I in we” notion which denotes an intersubjective perspective for explicating the development of self-concepts, and emphasizes that how people perceive themselves and how people evaluate their own efficacy to achieve in a given context are subject to the social recognition they receive (Su and Wong, 2022b). According to Honneth, people’s autonomy is not only personal but also social, and people’s exercises of personal and shared agency in a specific context are shaped by the level of social recognition that they can enjoy.

In the workplace context, social recognition is conceptually deemed important for exercising personal and shared agency of workers on both individual and team bases to enhance their workplace wellbeing and to co-construct an environment favorable to their sustainable career development for the following reasons. First, workers who are acknowledged by their supervisors and colleagues are more likely to self-recognize themselves, including recognizing their own existence, caring for their own needs, respecting their own opinions, and appreciating their own accomplishment and contribution in organizational contexts (Honneth, 2001, 2012). Second, workers with a high level of self-recognition may feel more secure to exercise their agency with regard to developing commitments and taking actions to achieve higher goals (Bandura, 2006, 2012). Third, workers being recognized are more likely to show their recognition to others and thus contribute to a group climate or synergetic atmosphere characterized by mutual recognition, and people working in such a group are more likely to make efforts to achieve common goals (Su et al., 2021a; Su and Wong, 2022a). In this connection, a decent work notion with a component of social recognition is expected to provide an intersubjective or interpersonal platform to foster the development of self-concepts in a positive and reciprocal manner, and promote the personal and shared agency of workers in reviewing the working conditions, enhancing their access to decent work, and fostering their workplace wellbeing and work fulfillment.

Moreover, a decent work notion incorporated with a social recognition component is aligned with the purpose to strengthen the epistemological ground underlying the psychology of working theory in relation to enhancing positive self-concepts for constructing meaning in work. Conceptually, the psychology of working theory takes social and economic constraints faced by people such as discrimination and marginalization encountered in the workplace as an important area for reform, suggesting that these constraints suffered by people are jeopardizing their development of psychological resources and depriving their access to decent work, which will further harm their satisfaction of needs, and achievement of wellbeing and self-fulfillment (Blustein et al., 2016, 2019; Duffy et al., 2016, 2019b; Pires et al., 2020; Autin et al., 2021; Masdonati et al., 2021). Access to decent work in both structural and dynamic senses is perceived by the psychology of working theory as the key mechanism for counteracting the negative effects caused by different sources of individual and social constraints on people’s wellbeing and self-fulfillment, as decent work is expected to provide some psychological conditions such as a sense of security or stability which are indispensable for cultivating workers’ positive self-concepts and satisfying their essential needs for survival, social connection, and self-determination (Blustein, 2013; Blustein et al., 2017). While being consistent with the psychology of working theory in enhancing positive self-concepts for constructing meaningful work, the recognition theory suggests an intersubjective perspective or the “I in we” notion as an alternative pathway for achieving these purposes. A decent work notion with a social recognition component can open a window for workers to find meanings on both individual and shared bases. In the past few years since 2017, the psychology of working theory has shown its great capacity to explicate the wellbeing and self-fulfillment of people in organizational contexts. To expand the decent work notion by incorporating a social recognition component is to broaden the psychology of working theory with a psychosocial perspective, which may enable this theory to promote positive changes through leveraging the interpersonal mechanism of social recognition situated within organizational contexts.

### The associations of decent work with job demands, job resources, and work engagement

Job demands and job resources are two overarching concepts that are widely used in vocational health psychology to describe the current job conditions of employees. Job demands refer to those physical, psychological, social, and organizational factors that cost energy for individuals to deal with, such as heavy workload and role conflict, whereas job resources denote the physical, psychological, social, and organizational factors that help individuals deal with these demands, such as social support, developmental opportunities, and job autonomy (Demerouti et al., 2001). Work engagement is defined as a fulfilling work-related state of workers encompassing three dimensions, namely vigor, dedication, and absorption (Schaufeli et al., 2002). Conceptually, the decent work notion discriminates from job demands, job resources, and work engagement for the following reasons. First, our decent work notion is situated in the psychology of working theory, whereas job demands, job resources, and work engagement are concepts developed in the field of vocational health psychology, which assumes that people working in a job that fits their attributes are more likely to enjoy better wellbeing and self-fulfillment (Autin and Duffy, 2019). According to this person-job fit perspective, those workers whose personal characteristics are in congruence with their job demands and job resources may show a high level of work engagement. Despite taking into consideration the influences of job conditions such as job demands and job resources on workers’ wellbeing (Bakker and Demerouti, 2007; Schaufeli and Taris, 2014), this approach is still criticized for underestimating the constraining effects of contextual and structural factors (Autin and Duffy, 2019). Living in a rapidly changing world, employees nowadays including social workers are faced with many contextual and structural constraints, such as climate change, work injustice, marginalization, overdemanding work, economic constraints, etc. that are harming their wellbeing and driving them to quit their jobs (Blustein et al., 2017; Golightley and Holloway, 2020; Agnimitra and Sharma, 2022; Su and Wong, 2022b). However, these constraints were often overlooked by existing studies on employees’ wellbeing, which were informed by the person-job-fit paradigm. Without addressing these contextual constraints, it is difficult to exercise individual and collective agency in organizational contexts for promoting the benefits of both employees and organizations. The rise of the psychology of working theory fills in this gap, as it addresses how contextual constraints are harming workers’ access to decent work and how workers may negotiate the constraints by exercising their own agency. Second, decent work is proposed by the ILO as a new lens for reviewing work conditions, which is enlisted as one of the important goals to call for collective actions for promoting global sustainable development (Bellace, 2011; Silva, 2021). The promotion of decent work around the world is following the Decent Work Agenda (DWA) with the explicit aims to realize four main values underlying the ILO’s actions, namely freedom, equity, security, and human dignity (International Labor Organization, 1999:3). Third, the decent work concept is characterized by a strong sense of future perspective, which is also the reason why decent work is suggested to be regarded as an aspired state of work (Webster et al., 2015).

Although situated in different conceptual frameworks, there may remain significant correlations of decent work with job demands, job resources, and work engagement, as these concepts are all concerned about work conditions or work-related state. As job resources and work engagement emphasize the positive elements of work, and job demands highlight the negative elements, we expect that the decent work notion incorporated with a social recognition component is to be positively associated with job resources and work engagement, and negatively associated with job demands. Moreover, according to the vocational health psychology, increased job resources can enhance work engagement of workers (Bakker and Demerouti, 2007; Schaufeli and Taris, 2014). Highlighting a future and intersubjective perspective for improving working conditions, decent work with a social recognition component is expected to add value to existing knowledge in terms of its positive effects on enhancing work engagement when controlling job resources.

### Striving for decent work among young adult social workers

Social workers as helping professionals are serving people in need and, in particular, those with vulnerabilities, including but not limited to children and youth at risk, older people living alone, people with physical or mental health challenges, etc. Studies alert us that emotionally demanding job nature and complex working conditions are associated with the negative states of wellbeing experienced by social workers, including burnout and turnover intention (Cho and Song, 2017; Su and Ng, 2019; Su, 2021). The negative wellbeing experienced by social workers may cause harm not only to social workers themselves but also to the organizations and the social work profession as a whole (Knight, 2013; Su et al., 2020a; Su, 2021). How to improve working conditions for enhancing employees’ wellbeing has become a pronounced challenge for organizations (Senreich et al., 2020; Gabriel and Aguinis, 2022). On the one hand, the negative wellbeing of social workers may reduce the efficacy of the organization; yet on the other hand, it is difficult for organizations to much reduce the workloads of their social worker employees for protecting their wellbeing, particularly in view of growing clientele and community needs in time of risks and uncertainties (Wong, 2015; Su et al., 2021b,d). In view that the decent work notion is promoted as a global sustainable development goal, it is deemed as a new lens to address these challenges to benefit both individual employees and organizations (Sheng and Zhou, 2021; Xu et al., 2022). Although the history of research on working conditions and workplace wellbeing of social workers has lasted for more than half a century, there is still no empirical study examining the working conditions of social workers through the lens of decent work or the framework of psychology of working theory. The manifestation of the decent work notion, antecedents, and outcomes of decent work in this profession are yet to be examined. In order to develop the social work profession and improve the working conditions of social workers in organizational contexts in a direction which is in line with the sustainable goal of “decent work for all” established by the United Nations (2015), it is worthwhile to strengthen the application of decent work notion informed by the psychology of working theory to this helping profession.

Theoretically, a decent work notion with a social recognition component is deemed important for social workers to develop their personal and shared identities at work and empower them to co-construct meaning in work for the following reasons. First, social work is a profession which is characterized by the co-construction of meaning in work through an intersubjective perspective. The working relationships developed by social workers with their service users, coworkers, and supervisors are all important for the former to assess whether their work is decent (Rollins, 2020; Su et al., 2021c). Second, social workers enjoying a higher level of social recognition at work may be more likely to develop a positive self-concept through their work, and find their work meaningful (Su et al., 2021b). In contrast, social workers working in a context where they are suffering from a low level of social recognition at work may question the meaning of their work, hold a low professional identity, and be more likely to quit their jobs (Niu and Haugen, 2019; Su et al., 2020b, 2021c; Su, 2021).

In the existing international dialog on developing decent work for constructing inclusionary employment, young people are considered to be in a more vulnerable position in view that they have relatively lower chances to take up a higher rank of job positions, and they are encountering more challenges in maintaining positive wellbeing and striving for career development as compared with those senior workers due to a lack of resources, opportunities, networks, and work experiences (Rosas and Rossignotti, 2005; Masdonati et al., 2020). In the social work profession, prior studies have found that young adult social workers earn less, undertake more frontline work and challenging direct service, and they are less likely to be engaged in organizational decision-making (Su and Ng, 2019; Su et al., 2020b, 2021c). So far, there are very few studies examining young adult social workers’ working conditions and wellbeing, and no empirical studies have reviewed the working conditions of young adult social workers through the lens of decent work. As young helping professionals are in a stage with a strong desire to strive for social recognition from others through excelling at work, the decent work notion with a social recognition component is considered important for expanding the concept of decent work in enhancing the capabilities and wellbeing of young adult social workers.

### The purposes, contexts, and hypotheses of this study

The first purpose of this study is to validate the factor structure of the Decent Work Scale incorporated with a social recognition component among young adult social workers aged 21–29 in Hong Kong as a developed economy, a special administrative region of China. Perceiving that it is a global trend to consider social recognition as a component of aspired work conditions, this study takes the initial steps to validate the decent work notion incorporated with social recognition in Hong Kong where East meets West, which is characterized by a fusion of both individualistic and collective cultures (Wagner and Moch, 1986; Lodge and Vogel, 1987; Zhang et al., 2022). As one of the most developed societies in Asia with a much longer history of social work development and professionalization which can be traced back to the 1960s (Lai and Chan, 2009; Su, 2021), Hong Kong seems to enjoy more resources and a more advantageous position to promote the decent work agenda for the social work profession.

The second purpose of this study is to test the discriminant validity of the Decent Work Scale incorporated with a social recognition component with some concepts related to social workers’ working conditions and wellbeing, namely job demands, job resources, and work engagement. Informed by the conceptualization of these concepts, the following hypotheses were formulated:

*H*1: The decent work notion incorporated with a social recognition component discriminates from the concept of job demands, job resources, and work engagement;

*H*2: The decent work notion incorporated with a social recognition component is positively associated with job resources and work engagement among social workers;

*H*3: The decent work notion incorporated with a social recognition component is negatively associated with job demands among social workers.

*H*4: The decent work notion incorporated with a social recognition component adds value to enhancing work engagement of social workers when controlling for the effects of job resources.

## Materials and methods

### Participants

Table 1 presents the sociodemographic information of participants who were all social workers aged 21–29, among which 72.9% were females and 27.1% were males. The gender imbalance of participants for this study was consistent with the fact revealed by a nationally representative sample of social workers in China (Su et al., 2020b). The mean age was 26.41 years (*SD* = 2.18) and 20.7% of participants had a master’s degree. 54.1% of the participants reported their job positions as junior rank and 44.5% as middle rank. The distribution of the job positions undertaken by young adult social workers in the current study was also consistent with that revealed in a large-scale study with a nationally representative sample of social workers in China (Su et al., 2020b). The monthly income of all participants was HK$14,999 or above, and the monthly income of most participants (60.5%) fell into the range of HK$20,000-HK$29,999, which is equivalent to that of US$2,600-US$3,899 (1HK$ ≈ 0.13 US$).

**Table 1:** Sociodemographic of participants (*N* = 362).

Variables	*N* (%)
*Gender*	
Female	264 (72.9)
Male	98 (27.1)
*Age*	
21	6 (1.7)
22	9 (2.5)
23	27 (7.5)
24	34 (9.4)
25	58 (16.0)
26	38 (10.5)
27	40 (11.0)
28	70 (19.3)
29	80 (22.1)
*Educational attainment*	
Secondary school or below	0
Associate degree	94 (26.0)
Bachelor’s degree	193 (53.3)
Master’s degree	75 (20.7)
*Marital status*	
Single or unmarried	306 (84.5)
Married or co-living	56 (15.5)
Divorced	0
*Position rank*	
Junior	196 (54.1)
Middle	161 (44.5)
Senior	4 (1.1)
CEO	1 (0.3)
*Monthly income (US$)*	
$1,949 or below	6 (1.7)
$1,950 – $2,599	14 (3.9)
$2,600 – $3,249	158 (43.6)
$3,250 – $3,899	61 (16.9)
$3,900 – $4,549	81 (22.4)
$4,550 – $5,199	33 (9.1)
$5,200 – $6,499	8 (2.2)
$6,500 – $7,799	1 (0.3)

### Data collection

Using cluster sampling procedures at organizational level, a cross-sectional study was conducted from June to December 2021. There are two types of organizations in Hong Kong employing social workers to provide social services, namely non-governmental organizations (NGOs) and the Social Welfare Department (SWD), one of the government departments of Hong Kong Special Administrative Region Government. Based on the membership list of NGOs available from the Hong Kong Council of Social Service, we categorized all NGOs into three types in accordance to the number of social workers employed, i.e., small- (1–30), medium- (31–99), and large- (≥100) size organizations. Through random sampling, 40% of each category of NGOs was randomly sampled as a pool for invitation for joining the study. At the same time, with the support of the biggest social workers association of those working in the Social Welfare Department, social worker members aged 21–29 of the association were invited to join the study. With the consent of sampled organizations, all their young adult social workers aged 21–29 were invited to fill in an online questionnaire survey *via* the Qualtrics system on an individual and voluntary basis. To ensure that the data collected from large-size NGOs would not dominate the results, a ceiling of 20 completed questionnaires at most was set for each of these NGOs. In total, we received 382 questionnaires and 362 were valid questionnaires without containing any missing data in the items measuring decent work. Prior ethical approval was granted by the research ethics committee of the university where the second author is affiliated with. Voluntary participation was ensured throughout the study and written informed consent was collected from each participant in the Qualtrics system in a confidential manner.

### Instruments

*Decent work* was measured by a new Decent Work Scale consisting of 15 items from the original Decent Work Scale developed by Duffy’s (Duffy et al., 2017) and three additional items derived from the Social Recognition Scale developed by Bjarnason (2015). Duffy’s Decent Work Scale consists of five factors (i.e., safe working conditions, access to health care, adequate compensation, free time and rest, and complementary values), and each factor was measured by three items. Responses to these 15 items were collected by a seven-point Likert-type scale ranging from strongly disagree (1) to strongly agree (7). There are four reversed items requiring recoding before calculating the sum of the score. The three items measuring social recognition were “I am encouraged to bring new ideas on how to do my job better,” “In the past weeks I have received praise for a job well done,” and “My supervisor, or someone at work, encourages my development.” Responses were collected by a five-point Likert-type scale ranging from strongly disagree (1) to strongly agree (5). In data analyses, we converted the responses to these three items from five-point to seven-point scale before calculating the sum of the total score, using the formula of *X*_7_ = (*X*_5_–1) * (6/4) + 1. The Cronbach’s alpha for the 18 items was 0.83.

*Job demands* were measured by the 19-item Job Demand Scale developed and validated among Chinese social workers (Su et al., 2021b) which consists of six factors, namely workload and cognitive demands, emotional demands, physical demands, work injustice and role conflict, role ambiguity, and job insecurity. Participants were asked to respond on a seven-point Likert-type scale from 1 (strongly disagree) to 7 (strongly agree). The Cronbach’s alpha for the Job Demands Scale was 0.84.

*Job resources* were measured by the 17-item Job Resources Scale developed and validated among Chinese social workers (Su et al., 2021b) which consists of five factors, namely autonomy, supervisor’s support, support from colleagues, learning opportunities, and job welfare. Participants were asked to respond to 17 items on a seven-point Likert-type scale from 1 (strongly disagree) to 7 (strongly agree). The Cronbach’s alpha for the Job Resources Scale was 0.91.

*Work engagement* was measured by the three-item Ultra-short Utrecht Work Engagement Scale validated by Su et al. (2022) for use in Chinese contexts, which was developed from the original nine-item Utrecht Work Engagement Scale (UWES-9; Schaufeli et al., 2006, 2019) and verified among Chinese social service workers (Fong and Ng, 2012). The UWES consists of three dimensions: vigor, dedication, and absorption, and the Ultra-short version uses one item measuring each dimension. Responses of the scale range from 0 (never) to 6 (every day). The higher sum score of the three items represents a higher level of work engagement. The Cronbach’s alpha for the three items was 0.85.

The instruments used in this study were all developed based on a psychological perspective which emphasizes how participants perceive their working conditions or work-related state, therefore the epistemological coherence among the instruments was ensured. All scales used were developed or validated in Chinese contexts except the decent work items. In view that the Chinese version of the 18 items for measuring decent work was not available when we designed this study, the translation and back-translation procedures were adopted for the purpose of cultural adaptation. The original scale in English was translated into Chinese by two bilingual researchers of the project for producing an initial translation, one is the co-investigator of the project and the other is a postgraduate research student. A back-translation of all the initial translation was produced by two native bilingual translators. A committee of all researchers chaired by the principal investigator of the project, who is the corresponding author of this article, reviewed all the translations and reached a consensus on all identified discrepancies. The pre-final version worked out by the committee was piloted with 20 social workers before collecting data for the study, who were not included as participants in the study implemented online later.

### Data analysis

Three steps of data analyses were performed. First, confirmatory factor analysis (CFA) was conducted by using the software of AMOS to verify the factor structure of the decent work scale. Three models were tested encompassing higher-order model, correlational model, and bifactor model. Second, we tested the bivariate associations of decent work with job demands, job resources, and work engagement among young adult social workers. Third, to examine the discriminant validity of the decent work scale, we drew the standardized loadings of items for the four study variables (i.e., job demands, job resources, decent work, and work engagement) in AMOS, conducted the average variance extracted (AVE) analyses based on the item loadings (Zaiţ and Bertea, 2011), and compared the square roots of AVE for all components of the decent work scale and their correlations with other variables. Finally, a hierarchical regression was tested to examine the value added by decent work for enhancing work engagement by controlling the effects of job resources.

## Results

**Figure 1:**
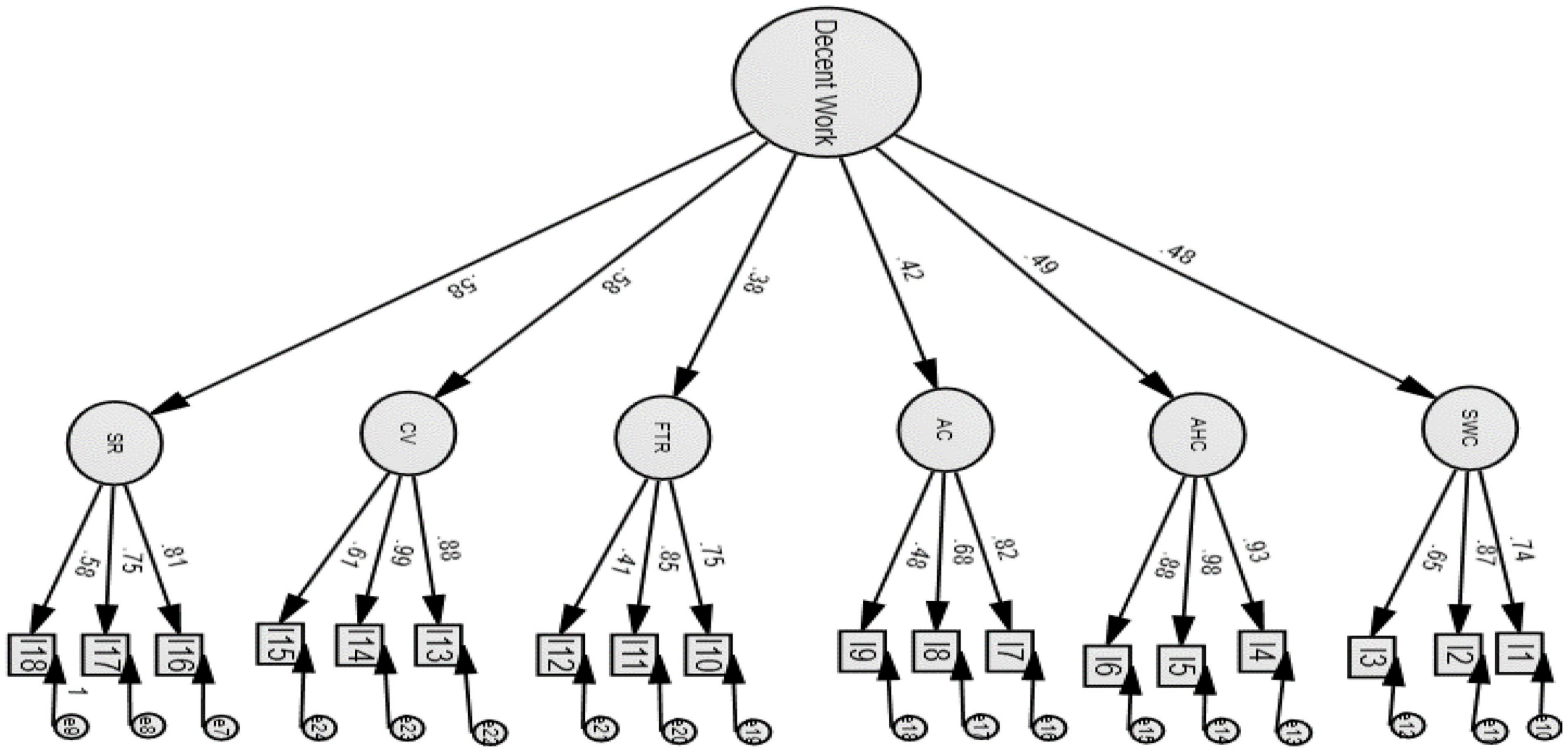
The higher-order model of the Decent Work Scale incorporated with a social recognition component among young adult social workers. All coefficients represent standardized estimates significant at 0.001 level; SWC, safe working conditions. AHC, access to health care. AC, adequate compensation. FTR, free time and rest. CV, complementary values. SR, social recognition.

**Figure 2:**
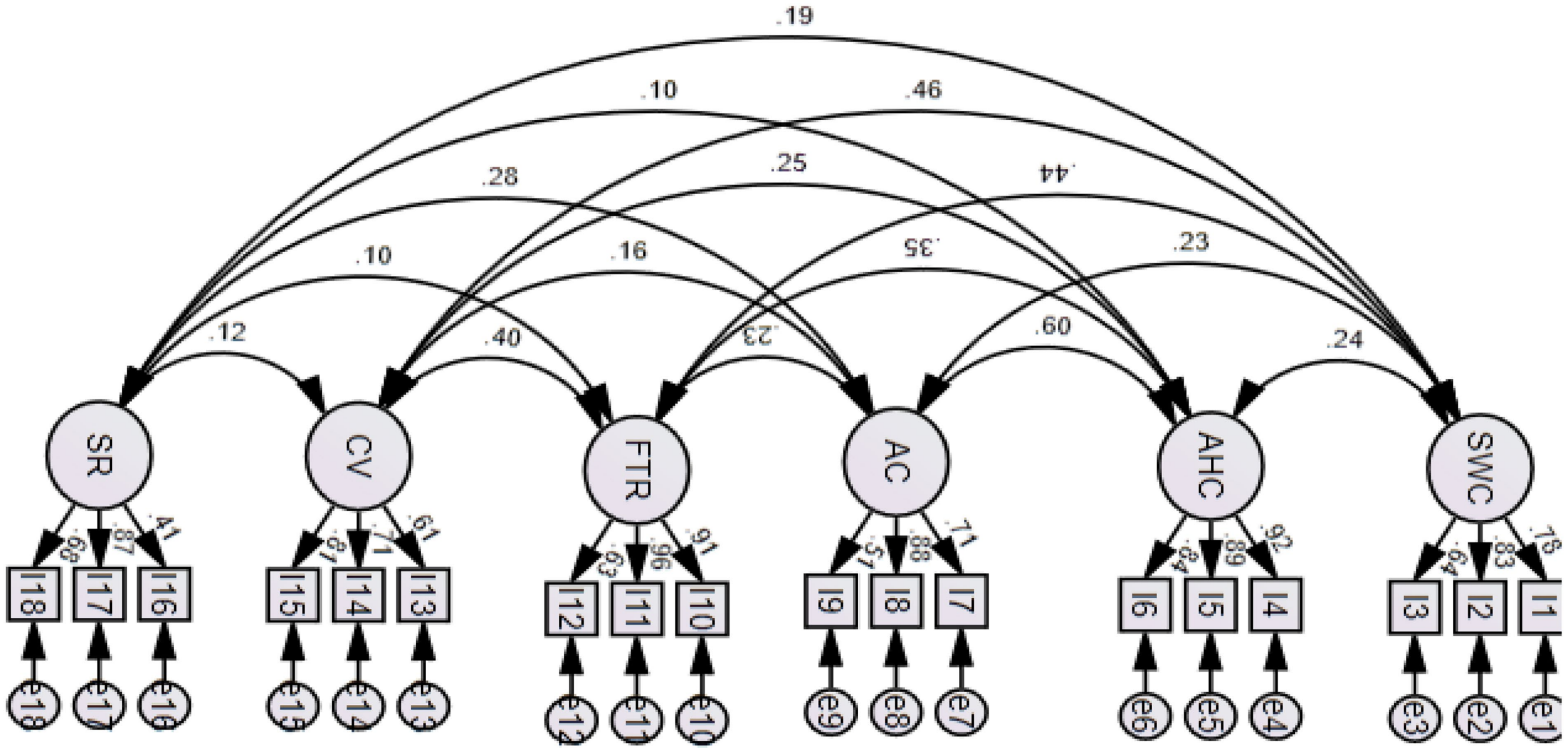
The correlational model of the Decent Work Scale incorporated with a social recognition component among young adult social workers. All coefficients represent standardized estimates significant at 0.001 level; SWC, safe working conditions. AHC, access to health care. AC, adequate compensation. FTR, free time and rest. CV, complementary values. SR, social recognition.

The results of CFA for the decent work scale with a workplace recognition component are presented in Table 2. Figure 1 presents the higher-order model of the Decent Work Scale with a social recognition component with the factor loadings of each factor falling into the range between 0.38 and 0.58. Figure 2 presents the six-factor correlational model and the correlations among the six factors range between 0.10 and 0.60. To determine the degree of model fit, we adopted a cluster of criteria on goodness-of-fit statistics: normed Chi-square (*X*^2^/df) <3, a CFI > 0.90, a Tucker–Lewis index (TLI) > 0.90, a RMSEA <0.08 (Hu and Bentler, 1998; Schermelleh-Engel et al., 2003). The CFA indexes for the higher-order model and the correlational model were both satisfactory as the RMSEA index was smaller than 0.08, and CFI and TLI were larger than 0.90. However, in view that the decent work notion was conceptually defined as an overarching concept comprising six factors and the correlation coefficients among some factors were lower than 0.30 in the correlational model, therefore, the higher-order model is concluded as a more preferred model.

**Table 2:** The results of CFA (*N* = 362).

Tested models	*p*	*X* ^2^	df	*X*^2^/df	CFI	TLI	RMSEA
Higher-order model^*^	0.000	300.25	126	2.38	0.95	0.93	0.062
Correlational model^*^	0.000	282.117	120	2.35	0.95	0.94	0.061
Bifactor model	0.000	1212.16	117	10.36	0.65	0.55	0.161

^*^Statistically acceptable models by referring to a cluster of criteria on goodness-of-fit statistics: normed Chi-square (X^2^/df) < 3, a CFI > 0.90, a Tucker–Lewis index (TLI) > 0.90, a RMSEA < 0.08 (Hu and Bentler, 1998; Schermelleh-Engel et al., 2003).

Table 3 displays the results regarding the correlations of decent work dimensions with validating scales (i.e., job demands, job resources, and work engagement) and the square root of average variance extracted (AVE) for all scales. The overall scale score of decent work correlated positively with job resources and work engagement, and negatively correlated with job demands, and thus the hypotheses regarding the correlations of the study variables were supported. All decent work components except the factor of free time and rest were significantly correlated in the expected directions with the validating scales; the component of free time and rest was not significantly correlated with work engagement. Moreover, the square roots of AVE for all components of the decent work scale and for the whole scale were larger than their correlations with the validating scales, which supported the discriminant validity of the decent work scale with a social recognition component.

**Table 3:** Square root of average variance extracted and correlations of decent work components with validating scales (*N* = 362).

**Variables**										
	1	2	3	4	5	6	7	8	9	10
1. SWC	0.76^#^									
2. AHC	0.24^***^	0.92^#^								
3. AC	0.18^**^	0.11^*^	0.67^#^							
4. FTR	0.23^***^	0.09	0.20^***^	0.72^#^						
5. CV	0.39^***^	0.35^***^	0.14^**^	0.23^***^	0.84^#^					
6. SR	0.37^***^	0.20^***^	0.16^**^	0.14^**^	0.32^***^	0.72^#^				
7. DW	0.66^***^	0.60^***^	0.48^***^	0.53^***^	0.68^***^	0.61^***^	0.78^#^			
8. JD	−0.45^***^	−0.26^***^	−0.27^***^	−0.50^***^	−0.34^***^	−0.34^***^	−0.60^***^	0.56^#^		
9. JR	0.52^***^	0.36^***^	0.29^***^	0.19^***^	0.44^***^	0.59^***^	0.66^***^	−0.43^***^	0.65^#^	
10. WE	0.32^***^	0.15^**^	0.12^*^	0.10	0.25^***^	0.37^***^	0.36^***^	−0.24^***^	0.41^***^	0.82^#^

SWC, safe working conditions. AHC, access to health care. AC, adequate compensation. FTR, free time and rest. CV, complementary values. SR, social recognition. DW, decent work. JD, job demands. JR, job resource. WE, work engagement.

* *p* < 0.05;

** *p* < 0.01;

*** *p* < 0.001.

# The numbers in the diagonal lines are the square roots of average variance extracted.

Table 4 presents the results of hierarchical regressions conducted in two studies for explaining the work engagement among social workers. The results showed that job resource and decent work were both favorable work conditions for enhancing work engagement in both studies and decent work with a social recognition component contributed a significant increase in the explained variances of work engagement in the tested models after controlling the effects of job resources. Therefore, the concurrent validity of the decent work notion was supported.

**Table 4:** Hierarchical regression analyses predicting work engagement (*N* = 362).

	Work engagement		
Predictors	Adjusted *R*^2^	*β*	*t*
Job resources	0.16^***^	0.32	4.95
Decent work with a recognition component	0.18^***^	0.15	2.24

^***^p < 0.001.

## Discussion

This study introduced how the decent work scale incorporated with a social recognition component was validated among young adult social workers in Hong Kong by using the data of a cross-sectional study. The findings can help expand the decent work notion, provide new directions for promoting and constructing decent work conditions, draw important implications for the development of the psychology of working theory, and inform the development of an enabling environment for enhancing the workplace wellbeing of young adult social workers and other helping professionals.

First, this is the first-ever study that focuses on developing the decent work notion with a social recognition component. The purpose of this study is consistent with the international calls for expanding the decent work notion and for considering social recognition as an important working condition of helping professionals (Chen, 2011; Charlesworth and Malone, 2017; Tiemeni, 2018; Molina et al., 2020). As decent work is proposed by ILO as a vehicle for social justice (1999, 2008), everyone deserves equal rights to access to a work with both stability and meaning. Yet it demands more efforts to enhance the inclusiveness of the current decent work notion and minimize constraints confronted by those marginalized populations in enjoying equal access to a decent work. In line with the recent findings of some studies (Di Fabio and Kenny, 2019; Masdonati et al., 2019; Ribeiro et al., 2019; Vignoli et al., 2020) conducted mainly in Western societies, which revealed that social recognition has the potential to become a decent work component, this study advances our understanding by providing the theoretical basis for incorporating a social recognition component into the decent work notion and by validating the new Decent Work Scale using quantitative data. The instrument used for measuring social recognition and the random sampling procedures used at organizational level in this study have also enhanced the rigor of the findings as compared with a relevant pioneering study conducted by Rossier and Ouedraogo (2021). The expanded notion of decent work supported by the current study provides new insights to inform future promotion and construction of decent work conditions in organizational contexts. Nowadays, various organizations including business organizations and NGOs are all facing many contextual constraints that are harming the wellbeing of employees as well as the sustainable development of the organizations. Those constraints may take place on the macrolevel such as pandemic crisis and post-pandemic recovery, and climate change (Golightley and Holloway, 2020) as well as on the interpersonal level such as discrimination and marginalization (Blustein et al., 2017, 2019; Duffy et al., 2019a). Decent work as a sustainable development goal is considered as an important way out to counteract the harmful effects of these constraints. The social recognition component supported by the current study as a part of the decent work scale provides a new psychosocial lens for reviewing working conditions, and for promoting decent work at interpersonal level in organizational contexts, which is deemed conducive to informing the design of strategies for human resources management and organizational development. Organizations may consider promoting decent work by modifying their human resources strategies to acknowledge diverse and distinct contributions made by their employees and enhance mutual recognition among their employees. Although the findings of the current study are confined to the Chinese context of Hong Kong, future studies to validate the new decent work scale may benefit more organizations and employees in other societies of the world.

Second, the decent work scale incorporated with a social recognition component enhances the capacity of the psychology of working theory for enabling people to improve their wellbeing and self-fulfillment in organizational contexts. The power of the psychology of working theory to promote the individual agency of workers in organizational contexts has been supported by empirical data in various studies (Duffy et al., 2020; Kim et al., 2020; Autin et al., 2021; Masdonati et al., 2021), yet the capacity of the theory is still undermined with respect to promoting the shared agency among workers and informing the development of relevant management practices and policies due to a weak link with an interpersonal perspective. As there are still so many political and socioeconomic barriers such as marginalization and discrimination affecting a large segment of population’s access to decent work, Blustein et al. (2019) suggested that expanding the decent work notion and enhancing the inclusiveness of the concept by incorporating new perspectives into the psychology of working theory are urgently needed. Recognition theory as an intersubjective perspective may address one of our essential needs of seeking social connections at work, provide an alternative pathway to counteract the negative impact of different barriers, and enable more marginalized people to access to a decent and meaningful work. Informed by Honneth’s recognition theory, this study takes the initiative to expand the decent work notion by incorporating a social recognition component for the sake of enhancing the psychosocial color of the psychology of working theory and enabling future applications of this theory for counteracting the negative effects of different constraints faced by workers and for making positive changes on both individual and interpersonal levels.

Third, this study reveals the associations of decent work with three important concepts widely used in vocational health psychology, namely job demands, job resources, and work engagement and the findings of this study also support decent work as a distinctive concept from job demands, job resources and work engagement. Consistent with the findings of recent studies (Kashyap et al., 2021; Xu et al., 2022) which revealed that decent work was positively associated with work engagement, this study advances our understanding regarding the relationships of decent work with a social recognition component with job demands, job resources, and work engagement. The discriminant validity of decent work in relation to these concepts can help differentiate the psychology of working theory from other perspectives in vocational health psychology. The value added by decent work with a social recognition component for enhancing work engagement after controlling the effects of job resources justifies the concurrent validity of the concept.

Fourth, this is the first study to introduce the decent work perspective into the social work profession which is expected to pave the way for future research and practice regarding reviewing the working conditions of social workers through the lens of the decent work notion and through the conceptual framework of psychology of working theory. In Hong Kong context, where there is a fusion of both individualistic and collectivistic cultures and the social work profession is more mature and publicly recognized, the higher-order model of the decent work scale with a social recognition component was revealed to be both statistically and conceptually acceptable. The findings considered favorable for applying the expanded notion of decent work incorporated with social recognition to the social work profession could help inform more theoretical and empirical work to further examine the working conditions of social workers with reference to the framework of psychology of working theory and promote more international and academic dialog about the application of the framework for studying and enhancing the workplace wellbeing and work fulfillment of other helping professionals, such as teachers, counselors, nurses, and domestic workers, etc.

Finally, this study has paid special attention to apply the decent work notion among young adult social workers who are perceived as less secure to achieve workplace wellbeing and career development when compared with their senior counterparts. Prior studies informed by the psychology of working theory revealed that young people are in a more marginalized position in the labor market and they have less access to decent work or more access to precarious work (Allan et al., 2020; Masdonati et al., 2020). The findings of the current study may provide a new direction to study wellbeing and self-fulfillment of young people in organizational contexts with the lens of decent work. When compared with their senior counterparts, young employees are largely disadvantaged in relation to access to decent work and positioned at a stage with strong desire for seeking recognition from others, and thus based on the findings of the current study, it is recommended to enhance young people’s access to decent work by expanding their sources and spheres of recognition (Su et al., 2021a; Su and Wong, 2022a,b); specifically, organizations may offer their young employees more opportunities to make contributions at work and acknowledge their diverse and distinct contributions in a concrete or explicit manner. The recognition received by young employees from their service users, coworkers, supervisors, human resources officers, etc. in different forms such as care, respect of rights and agency, encouragement, appreciation, etc. can be taken as decent work elements favorable for promoting their wellbeing and self-fulfillment. The findings of this study can direct more attention to review the working conditions of young adults by using the expanded notion of decent work and thus may provide mechanisms for enhancing their workplace wellbeing and career development.

## Limitations

The empirical data of this study are limited to young adult social workers in Hong Kong. There is also room to increase the sampling size of future studies, particularly when considering that the participants will be less occupied by increasing workload and pressure caused by the COVID-19 pandemic during which online data were collected. We cannot thus generalize the findings to mainland China or other Western societies. Future studies are also suggested to validate the newly validated decent work scale among social workers of different age groups, and validate the scale in different helping professions and different youth groups in other societies of diverse cultural backgrounds. Moreover, although cluster sampling procedures used at organizational level contributed to the rigor of the current study, simple random sampling at both organizational and individual levels used in future studies is needed to further reduce bias of the findings.

## Conclusion

To conclude, the results of the current study suggested that social recognition is a relevant dimension for analyzing decent work as the psychometric properties including the excellent reliability, the six-factor-higher-order model, the discriminant validity, and concurrent validity of the Decent Work Scale incorporated with a social recognition component. The findings of this study support the future use of the Decent Work Scale incorporated with a social recognition component for reviewing and developing working conditions and interpersonal mechanisms favorable for achieving wellbeing and work fulfillment among young adult social workers in Hong Kong. The findings of this study support the expanded notion of decent work incorporated with the psychosocial component of social recognition, which deserves further research studies for reviewing and informing the career development of helping professionals and service workers in diverse organizational settings.

## Data availability statement

The raw data supporting the conclusions of this article will be made available by the corresponding authors upon reasonable request, without undue reservation.

## Ethics statement

This study involving human participants was reviewed and approved by the Research Ethics Committee of Hong Kong Baptist University. The participants provided their written informed consent to participate in this study.

## Author contributions

XS was responsible for designing the study, conducting data collection and data analysis, and drafting and revising the article. VW was responsible for co-designing the study, conducting data collection, and drafting and revising the article. KL was responsible for co-designing the study and data collection. All authors contributed to the article and approved the submitted version.

## Funding

This work was supported by the Research Grants Council of Hong Kong (HKBU/GRF/12600819).

## Conflict of interest

The authors declare that the research was conducted in the absence of any commercial or financial relationships that could be construed as a potential conflict of interest.

## Publisher’s note

All claims expressed in this article are solely those of the authors and do not necessarily represent those of their affiliated organizations, or those of the publisher, the editors and the reviewers. Any product that may be evaluated in this article, or claim that may be made by its manufacturer, is not guaranteed or endorsed by the publisher.
